# Evaluation of resistance of human head lice to pyrethroid insecticides: A meta-analysis study

**DOI:** 10.1016/j.heliyon.2023.e17219

**Published:** 2023-06-19

**Authors:** Ebrahim Abbasi, Salman Daliri, Zahra Yazdani, Shokrollah Mohseni, Ghulamraza Mohammadyan, Seyedeh Niloofar Seyed Hosseini, Reza Nasiri Haghighi

**Affiliations:** a Research Center for Health Sciences, Institute of Health, Shiraz University of Medical Sciences, Shiraz, Iran; b Dept. of Biology and Control of Disease Vectors, School of Health, Shiraz University of Medical Sciences, Shiraz, Iran; c Department of Biology, College of Sciences, Shiraz University, Shiraz, Iran; d Research Clinical Research Development Unit, Imam Hossein Hospital, Shahroud University of Medical Sciences, Shahroud, Iran

**Keywords:** Human head lice, Resistance, Insecticides, Pyrethroid, Meta-analysis

## Abstract

**Introduction:**

Pediculosis is one of the most common annoying infections caused by parasitic lice in humans. Pyrethroids are one of the main insecticides used to treat this infection. But recently, due to the Resistance of lice to this group of insecticides, its insecticidal effects have been affected. The present study was conducted through a meta-analysis to investigate the prevalence of pyrethroid resistance against these insecticides worldwide.

**Methods:**

This study was conducted as a meta-analysis of the prevalence of treatment resistance in human head lice against pyrethroid insecticides worldwide. Based on this, all articles published without a time limit until the end of June 2022 in PubMed/MEDLINE, Web of Science (ISI), Scopus, and Google Scholar databases were extracted and using random-effects meta-analysis model statistical methods in the meta-analysis, Cochrane, Index I^2,^ and funnel plot were analyzed by STATA software.

**Results:**

Twenty studies were included in the meta-analysis process. According to this, the prevalence of pyrethroid resistance insecticides in human head lice was estimated at 59% (CI95%: 50%–68%). Among pyrethroid insecticides, the highest prevalence of pyrethroid resistance against permethrin insecticide was 65%. Regarding the prevalence of Resistance by year, the prevalence before 2004 was estimated at 33%, but after 2015, this rate reached 82%. Also, the majority of pyrethroid resistance was estimated at 68% using genetic diagnosis methods and 43% using clinical diagnosis methods.

**Conclusion:**

More than half of human head lice pyrethroid resistance insecticides. Based on this, it is recommended that before using this treatment method to treat human head lice Infestation, it should investigate the prevalence of pyrethroid resistance in that area, and if the majority of Resistance is high, alternative or combined treatment methods should be used.

## 1 Introduction

Pediculus humanus capitis or human head louse, is a parasitic insect caused by a hematophagous ectoparasite that lives in human hair and feeds on human blood. Infestation with Pediculus humanus capitis is often asymptomatic and not pathogenic for humans, but sometimes it causes skin irritation [1,2]. But the importance of this pollution is its widespread prevalence. In recent decades, most of the world’s countries in North America, South America, Australia, Europe, and Asia have been involved in this pollution, and its prevalence is increasing [[3], [4], [5], [6]]. As a result, controlling and preventing the spread of this Infestation is very important. The transmission of this Infestation is from person to person, so in case of contact with an infected person, especially in crowded places, it is transmitted to other people and causes them to become infected [7,8]. Treating infected people is one of the most critical approaches to preventing and controlling Pediculosis. Because when the source of infestation is controlled, the disease will no longer be transmitted. One of the most essential and standard treatment methods is using safe insecticides. Today, various insecticides are used worldwide to treat Pediculosis. Still, the most common insecticide group is Pyrethroids, sold and used under the trade names Permethrin, Phenothrin, and Pyrethrin [[9], [10], [11]]. This group of insecticides causes their death by disrupting the nervous system of lice. But recently, due to indiscriminate use and incomplete Treatment, lice have become resistant to this group of insecticides. Due to the lack of complete Treatment of the source of Infestation, its prevalence is increasing [[12], [13], [14]].

Studies show that permethrin’s effectiveness in treating Pediculosis has decreased from 97% and phenothrin from 75% to less than 15%. The subject indicates that using this insecticide group is ineffective [10,11,15]. In lice, there are different mechanisms for pyrethroid resistance against insecticides. The primary tool, especially against pyrethroid insecticides, is Resistance at the target site, created by point mutations. It makes the real targets of an insecticide less sensitive to the active ingredient [16]. This mutation in the voltage-sensitive sodium channel gene (Vssc) leads to Resistance in sodium channels [[17], [18], [19]]. By acting on sodium channels, pyrethroid insecticides interfere with the opening and closing of these channels, and this action leads to paralysis and, eventually, the death of lice. But when these mutations occur, the sensitivity of lice to insecticides is reduced, and the Treatment with them will be incomplete or ineffective [13,19,20]. As a result, it is essential to be aware of the prevalence of Resistance to insecticides to adopt the appropriate Treatment. There are various methods to detect the majority of Resistance in lice. The simplest way is to use insecticides to kill live lice and observe the proportion of live lice (clinical Method). The following Method uses molecular indicators to determine the presence of treatment-resistant genes in lice (genetically Method). In this Method, the gene mutation pyrethroid resistance and its ratio in lice are determined after extracting the genes in the laboratory environment. It includes Polymerase Chain Reaction (PCR), Quantitative Sequencing (QS), and Serial Invasive. Signal Amplification Reaction (SISAR) Protocol [17,21].

In general, it is necessary to be aware of the prevalence of treatment resistance in lice against pyrethroid insecticides to adopt the appropriate Treatment. Based on this, the present study was conducted to determine the prevalence of treatment resistance in human head lice against pyrethroid insecticides via systematic review and meta-analysis worldwide.

## 2 Material and methods

### 2.1 Protocol

The Preferred Reporting Items conducted this systematic review and meta-analysis study for Systematic Reviews and Meta-Analyses (PRISMA) statement [22]. This study aimed to estimate the prevalence of Resistance to human head lice treatment against pyrethroid insecticides.

### 2.2 Search strategy and study selection

The search for articles on the prevalence of pyrethroid resistance of human head lice against pyrethroid insecticides was conducted in the scientific databases PubMed/MEDLINE, Web of Science (ISI), Scopus, and Google Scholar without a time limit until the end of June 2022. It used the collection of related keywords extracted from medical subject headings (MeSH), including head lice, human head lice, Pediculosis, Pediculosis capitis, Human Head Louse, Insecticide, Pediculicide, resistant, Pyrethroids, pyrethrins, permethrin, phenothrin, Treatment. These keywords were searched using AND and OR operators in singular and compound forms in titles, abstracts, and keywords. It also manually checked the reference lists of related articles for other possibly relevant articles not found through the electronic search strategy. Searching and extraction of articles were done independently by two trained authors in the field of searching articles in the medical information database.

### 2.3 Inclusion and exclusion criteria

All English language articles published in human head louse pyrethroid resistance, which investigated the pyrethroid resistance insecticides and the ratio of Resistance reported in them and were of good quality, were included in the study. Articles with low quality, studies conducted on other insects except for human head lice, non-reporting of quantitative pyrethroid resistance, reviews of other insecticides, qualitative studies, case reports, case series, reviews, and meta-analyses were excluded from the study.

### 2.4 Quality assessment

It used the Strobe checklist (Strengthening the Reporting of Observational Studies in Epidemiology) to evaluate the quality of the articles [23]. This checklist has 22 sections in the fields of title, abstract, introduction, objectives, study method, Inclusion and exclusion criteria, sample size, study group, results, and limitations, which are scored based on the importance of each section. Be. The minimum and maximum scores obtained by this checklist are 15 and 33. In this study, the minimum acceptable score was 20 [24].

### 2.5 Data extraction

Two authors independently extracted the required information mentioned below from the articles: name of the author, year of study, place of study, sample size, type of insecticide used for Treatment, Method of detecting Resistance, and prevalence of Resistance.

**Fig. 1:**
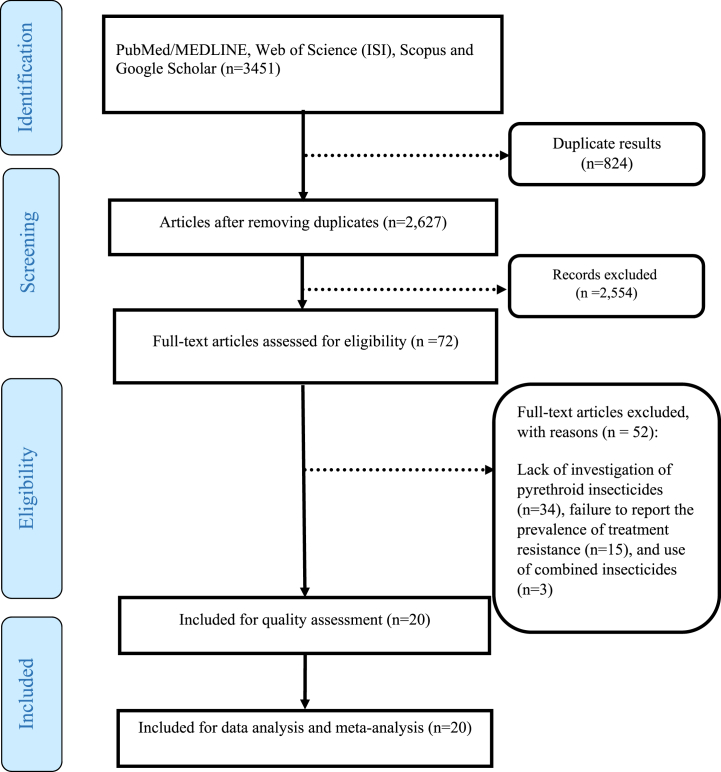
The PRISMA flow diagram.

According to the PRISMA flow chart (Fig. 1), 3451 studies were extracted during the search in scientific databases. Out of this number, it removed 824 studies due to repetition and from the remaining 2627 articles. After reviewing the title and abstract, it removed 2554 pieces due to a lack of relevance. Then it studied the full text of the remaining 72 articles. Of these, 52 articles were excluded for the following reasons: lack of investigation of pyrethroid insecticides (n = 34), failure to report the prevalence of treatment resistance (n = 15), and use of combined insecticides (n = 3). Finally, 20 studies met the eligibility criteria and underwent systematic review and meta-analysis.

### 2.6 Statistical analysis

Random-effects meta-analysis model with a 95% confidence interval was used to compare the prevalence of Resistance. Subgroups were analyzed based on the type of Treatment, diagnosis, Method, and year of study. The I2 test was used. In the results, 0%–25% heterogeneity was considered low, 25%–50% as a medium, and 50%–75% as high heterogeneity to evaluate the heterogeneity between the selected studies. Publication bias was assessed by Begg-Mazumdar and Egger tests. In addition, the subject performed a sensitivity analysis to determine the stability of the results. P-values above 0.05 indicate that the total variance is due to within-study conflict rather than between. Stata V.14 (Stata Corp.) was used for statistical analysis.

## 3 Result

Twenty studies conducted between 1991 and 2020 with a sample size of 24,651 human head lice were included in the meta-analysis process. The highest prevalence of human head lice resistance against pyrethroid insecticides was 99%, and the lowest majority was 5.4%. The characteristic of the reviewed articles is presented in Table 1.

**Table 1:** Characteristic of the articles included in the systematic review.

Author	Place of study	Year of Study	Sample size	Resistance Prevalence	Quality assessment
Mumcuoglu KY [25]	Israel	1991	50	21.2	Mild
Thomas D [26]	United Kingdom	2006	316	5.4	High
Burgess IF [11]	United Kingdom	2013	47	85.1	Mild
Kim HJ [21]	USA	2004	27	56	Mild
Kasai SH [27]	Japan	2009	630	9	High
Yoon KS [28]	Netherlands and United States	2014	33	99	High
Bouvresse S [29]	France	2012	670	98	High
Kasai SH [30]	Japan	2009	282	9	Mild
Eremeeva ME [14]	USA	2017	259	99	High
Yoon KS [31]	North American	2014	480	99	High
Audino G [32]	Argentina	2005	240	36	Mild
Gao JR [33]	USA	2003	339	41	High
Firooziyan S [19]	Iran	2017	2765	59	High
Durand R [34]	France	2011	1551	93	High
Toloza AC [35]	Argentina	2014	154	88	High
Roca-Acevedo G [36]	Chile	2019	99	49	Mild
Ponce-Garcia G [37]	Mexico	2017	468	62	High
Gellatly KJ [38]	USA	2016	14,281	98	High
Karakuş M [39]	Turkey	2020	1767	99	High
Chosidow O [40]	New Zealand	1994	193	60	Mild

**Fig. 2:**
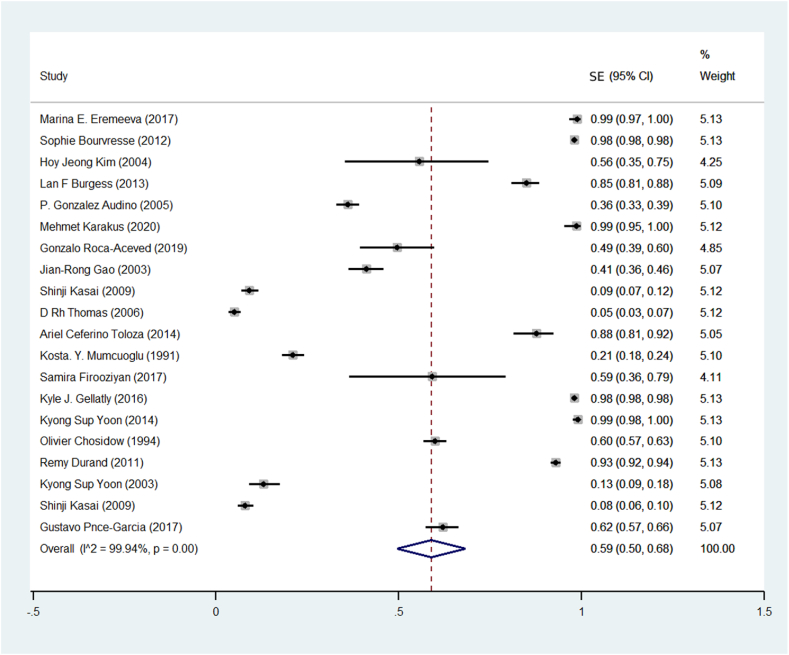
Pooled prevalence rate of pyrethroid resistance human head lice against pyrethroid insecticides based on random-effects meta-analysis model. The midpoint of each line segment shows the prevalence estimate, the length of the line segment indicates the 95% confidence interval in each study, and the diamond mark illustrates the pooled Resistance prevalence.

**Fig. 3:**
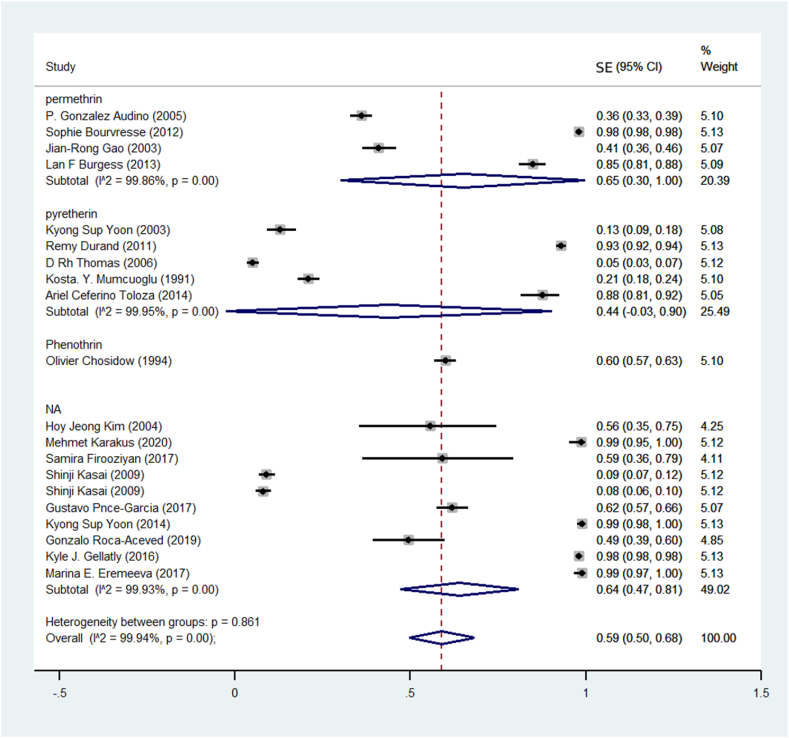
Pooled prevalence rate of pyrethroid resistance human head lice based on the type of insecticide using a random-effects model.

Based on the findings of the meta-analysis of 20 studies, the prevalence of Resistance to pyrethroid insecticides in human head lice was estimated at 59% (CI95%: 50%–68%) (Fig. 2). Among pyrethroid insecticides, the highest prevalence of pyrethroid resistance against permethrin insecticide was 65% (Fig. 3).

**Fig. 4:**
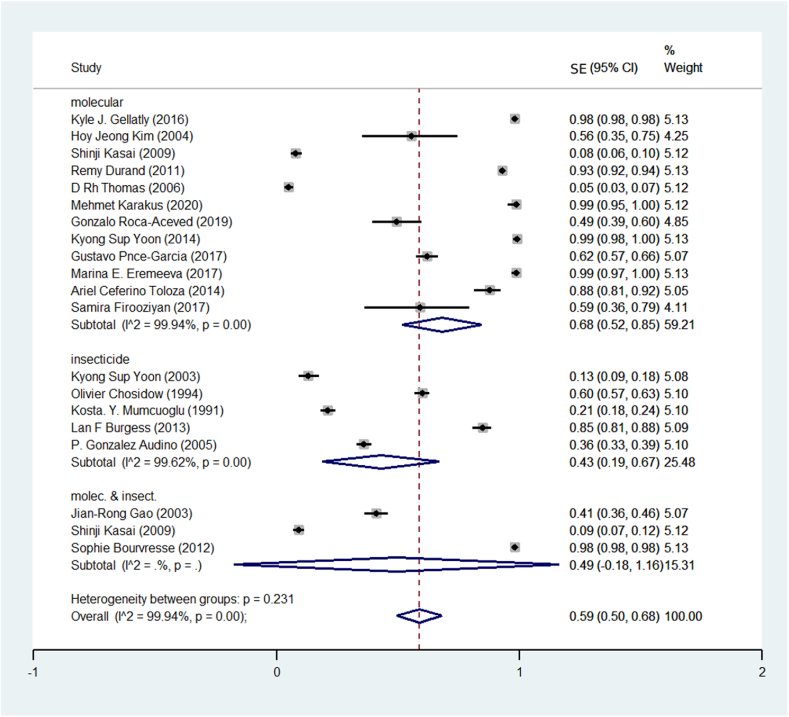
Pooled prevalence rate of pyrethroid resistance human head lice based on the Method for diagnosing Resistance using a random-effects model.

**Fig. 5:**
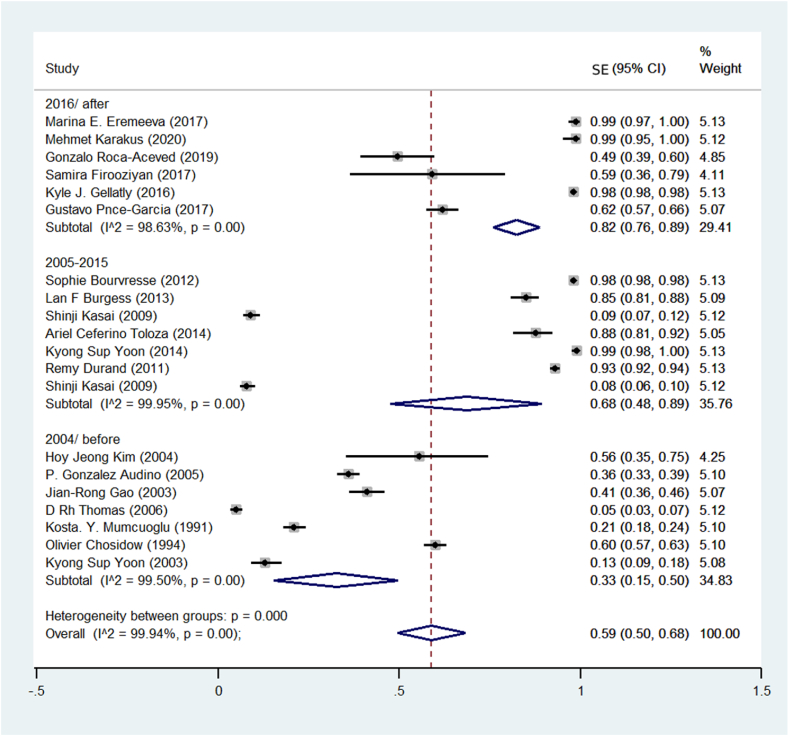
Pooled prevalence rate of pyrethroid resistance head lice treatment based on the year of the study using the random-effects model.

They used molecular methods to detect pyrethroid resistance in human head lice. They also used the death rate of head lice after Treatment. Based on this, the meta-analysis results showed that in the studies that used the genetic diagnostic Method, the prevalence of pyrethroid resistance was 68%. In the studies that used the clinical Method (rate lice live after using insecticide), the majority of Resistance was 43%. In the studies that used both methods, the prevalence of pyrethroid resistance was estimated at 49% (Fig. 4). The studies were divided into three 10-year periods. It includes before 1383, from 1384 to 1394, and after 1394 Regarding the prevalence of pyrethroid resistance. Fig. 5 shows the findings of the meta-analysis of studies. The majority of treatment resistance was estimated before 2004 (33%), between 2005 and 2015 (68%), and after 2015 (82%).

**Fig. 6:**
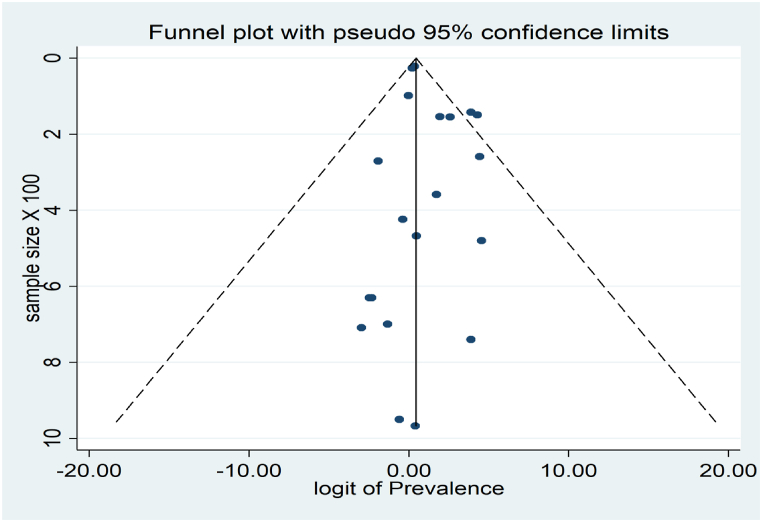
Funnel plot of the prevalence of human head lice resistance.

Funnel plot, Begg’s, and Egger’s tests were used to investigate the Publication bias. The symmetry of the articles in the funnel plot indicates that publication bias has not occurred (Fig. 6). Also, the results of Begg’s and Egger’s tests show no publication bias in this meta-analysis. The result of Begg’s test was Z = −0.39 and P = 0.75, and the development of Egger’s test was Z = 1.60 and P = 0.11.

## 4 Discussion

The present study’s findings showed that more than half of human head lice are resistant to pyrethroid insecticides, and the highest prevalence of Resistance to permethrin insecticide was observed. Also, the majority of pyrethroid resistance has increased during the past three decades, in the study by Larkin et al. (2020) [41] in Honduras, 93.9. (The statement needs to be short). In the study by Brownell et al. (2020) [42] in Thailand, 40%, and in the study of Roca Acevedo et al. (2019) [36] in Chile, 50% of head lice had a mutation in the T917I allele, which indicates pyrethroid resistance against pyrethroid. Ponce et al. (2017) reported in Mexico that 78.2% of the samples had a mutation in the T929I allele, which leads to pyrethroid resistance against pyrethroid insecticides [37]. In the study by Gellatly et al. (2016), 98.3% of the lice studied in the United States had an allele resistant to pyrethroid treatment [38]. Yoon et al. (2014) investigated the trend of Resistance for ten years in North America. Based on the findings, between 1999 and 2009, the prevalence of pyrethroid resistance of Pyrethroids was estimated at 84.4%. This proportion was reported as 97.1% in 2008 and 99.6% between 2007 and 2009, which indicates the increasing trend of treatment resistance [31]. In Mohammadi et al. in the metaanalysis study (2021), the prevalence of pyrethroid resistance in human head louse was estimated at 76.9%, which indicates that a high proportion of lice are resistant to permethrin [43]. Kasai et al. (2009) stated that Japan’s large head lice population is resistant to pyrethroid [27]. Cueto et al. (2008) reported Resistance to permethrin in embryos and lice eggs in Argentina [44]. In the study of Kristensen et al. (2006) in Denmark, the study of head lice showed that the genetic mutation of alleles T929I and L932 was present in them and caused Resistance to permethrin and Malathion [45]. Yoon et al. (2004) observed Resistance to permethrin and Malathion in head lice in California and Florida [46].

Gao et al. (2003) reported Resistance to permethrin caused by T929I and L932F alleles in California, Texas, and Florida [33]. Lee et al. (2000) registered Resistance to permethrin and Malathion in England [47]. Durand et al. (2014) in France, considering the high rate of head lice genotypic Resistance against pyrethroid insecticides, did not find its use effective for treating head lice and recommended alternative treatment methods [34]. Lebwohl et al. (2007), who investigated the Treatment of Pediculosis, considering the 12-day period for head lice to mature, recommended using pediculicides that do not have ovulatory properties (such as Pyrethroids). The Treatment should be carried out in 2 or 3 periods. Treatment with pediculicide against which there is genetic Resistance is ineffective [48]. Ebrahimi et al. (2021) in Iran identified three kdr point mutations in amino acids D820E, L840F, and N874G, corresponding to the replacement of aspartic acid to glutamic acid, leucine to phenylalanine, and asparagine in the human head louse population, which L840F was identified as a new mutation [49]. Ghavami et al. (2023) reported the presence of M815I, T917I and L920F mutations in 91.6, 4.2 and 4.2% of human head lice samples, respectively [50].

The survival of head lice after using insecticides can have various reasons, including the patient’s non-adherence to the treatment protocol and incorrect Treatment (low dose or incorrect use), lack of egg-laying properties, re-infestation, and Resistance against Treatment. Excessive pressure in the widespread and incomplete use of insecticides to control lice is the leading cause of increasing pyrethroid resistance in human head lice. Resistance to insecticides leads to an increase in the prevalence and chronicity of infestation in people, followed by the rise in treatment costs, the ineffectiveness of insecticides, the use of additional treatments, and an increase in possible toxicity and discomfort for infested people [51]. Based on this, choosing the suitable insecticide and the correct prescription and Treatment is essential in controlling the spread of human head lice and reducing the prevalence of pyrethroid resistance.

Different methods are used to determine the proportion of pyrethroid resistance in lice, which include: 1- Diagnosis of clinical Resistance, in the form of the balance of surviving lice one day after the use of insecticide, 2- Diagnosis of parasitic Resistance, in the form of evaluation of the resistance proportion of lice outside the human body to pediculicide compounds and 3- diagnosis of genetic Resistance, assessment in the form of evaluation of polymorphism in genes related to Resistance in lice. The present study’s findings showed that the prevalence of Resistance using genetic diagnostic methods was higher than the prevalence using the proportion of dead lice after using insecticides. One of the reasons for its decrease can be incorrect Treatment, such as low dose of insecticide or inaccurate use of medicine and lack of ovulatory properties. For genetic diagnosis, Karakuş et al. (2020) use the polymerase chain reaction (PCR) method to screen the mutation of resistant alleles in lice, which requires small amounts of DNA for analysis and is easy to perform in a laboratory environment recommended [14,18,39]. Lee et al. recommended the Quantitative Sequencing (QS) method for screening the mutation of treatment-resistant alleles in head lice due to its speed, accuracy, simplicity, and widespread use [52]. Kim et al. in Argentina recommended using serial invasive signal amplification reaction (SISAR) protocol to screen and diagnose treatment resistance [21]. Although genetic methods are more accurate for diagnosing pyrethroid resistance, clinical practices are also suitable due to their availability, ease, and cheapness.

### 4.1 Limitations

The limitations of the present study include 1- the uncertainty of the type of insecticide used in some studies and 2- conducting studies in different years and countries, which may have different treatment approaches. 3- The existence of heterogeneity between studies.

## 5 Conclusion

The present study’s findings showed that more than half of human head lice are resistant to Treatment with pyrethroid insecticides. Based on this, widespread use without specific treatment resistance in infected areas is ineffective. Based on this, it is recommended that before using this treatment method to treat human head lice Infestation, it should investigate the prevalence of pyrethroid resistance in that area. Suppose the majority of Resistance is high. It should use alternative or combined treatment methods. Study systematically, and considering that it registered its search strategies in the Prospero system, it is easily repeatable. Its results can be feedback for evaluating human head lice control products and improving pediculosis control strategies.

## Declaration

### Ethics approval and consent to participate

Not applicable.

### Availability of data and materials

All the data obtained from this study are included in the text of the article.

### Funding

This research received no specific grant from any funding agency in the public, commercial, or not-for-profit sectors.

### Authors' contributions

EA determined the title, wrote and registered the protocol, and submitted the article. EA and SM extracted the files from the databases. ZY, SH, and RN, screening, and selection of final reports. GM and Abbasi, data extraction. SD wrote the article. All authors read and approved the final manuscript.

## Declaration of competing interest

The authors declare that they have no known competing financial interests or personal relationships that could have appeared to influence the work reported in this paper
